# Docosahexaenoic Acid Inhibits PTP1B Phosphatase and the Viability of MCF-7 Breast Cancer Cells

**DOI:** 10.3390/nu11112554

**Published:** 2019-10-23

**Authors:** Alicja Kuban-Jankowska, Magdalena Gorska-Ponikowska, Kamlesh Kumar Sahu, Tomasz Kostrzewa, Michal Wozniak, Jack Tuszynski

**Affiliations:** 1 Department of Medical Chemistry, Medical University of Gdansk, 1 Debinki St., 80-211 Gdansk, Poland; m.gorska@gumed.edu.pl (M.G.-P.); tomasz.kostrzewa@gumed.edu.pl (T.K.); mwozniak@gumed.edu.pl (M.W.); 2 Institute of Biomaterials and Biomolecular Systems, Department of Biophysics, University of Stuttgart, 70550 Stuttgart, Germany; 3 The Euro-Mediterranean Institute of Science and Technology, 90127 Palermo, Italy; 4 Li Ka Shing Applied Virology Institute, Department of Medical Microbiology and Immunology 6-020 Katz Group Centre, University of Alberta, Edmonton, AB T6G 2E1, Canada; ksahu@ualberta.ca; 5 Department of Oncology, University of Alberta, Edmonton, AB T6G 1Z2, Canada; jack.tuszynski@gmail.com; 6 Department of Physics, CCIS, University of Alberta, Edmonton, AB T6G 2E1, Canada; 7 DIMEAS, Politecnico di Torino, Corso Duca degli Abruzzi, 24, 10129 Torino, Italy

**Keywords:** breast cancer, docosahexaenoic acid, omega-3 acids, protein tyrosine phosphatase PTP1B

## Abstract

Background: Docosahexaenoic acid (DHA) is an essential polyunsaturated fatty acid compound present in deep water fishes and dietary supplements, with a wide spectrum of potential health benefits, ranging from neurological to anti-inflammatory. Methods: Due to the fact that DHA is considered a breast cancer risk reducer, we examined the impact of DHA on MCF-7 breast cancer cells’ viability and its inhibitory properties on protein tyrosine phosphatase 1B (PTP1B), a pro-oncogenic phosphatase. Results: We found that DHA is able to lower both the enzymatic activity of PTP1B phosphatase and the viability of MCF-7 breast cancer cells. We showed that unsaturated DHA possesses a significantly higher inhibitory activity toward PTP1B in comparison to similar fatty acids. We also performed a computational analysis of DHA binding to PTP1B and discovered that it is able to bind to an allosteric binding site. Conclusions: Utilizing both a recombinant enzyme and cellular models, we demonstrated that DHA can be considered a potential pharmacological agent for the prevention of breast cancer.

## 1. Introduction

Breast cancer is a common malignant female cancer affecting women worldwide. Its therapy involves surgical intervention and radiotherapy completed with adjuvant chemotherapy. The development of breast cancer is associated with numerous disorders of tyrosine phosphorylation pathways [1]. 

Protein tyrosine phosphatase 1B (PTP1B) is involved in the dephosphorylation process of tyrosine kinases responsible for breast cancer development, i.e., HER1/EGFR, Src, JAK, as well as of signal transducer and activator of transcription (STAT). PTP1B was found to be overexpressed in breast cancer cells and trigger the tumor growth [2]. 

PTP1B is considered a potentially important target for the treatment or prevention of breast cancer. Targeting PTP1B may be effective in breast cancer prevention; however, it is not clear if it could be effective in the treatment of advanced breast cancers of the HER2-positive subtype [3]. It has been discovered that whole-body deletion of PTP1B in mice delays or protects against HER2/Neu-induced mammary carcinogenesis [4]. In contrast, overexpression of PTP1B in the mammary gland leads to spontaneous breast cancer development. The control of ErbB2-induced mammary tumorigenesis by PTB1B is regulated through the attenuation of both MAP kinase (MAPK) and Akt pathways [4]. These findings support the hypothesis of PTP1B being a new therapeutic target in breast cancer.

Inhibitors of PTP1B phosphatase can also be promising compounds for treating metabolic diseases, i.e., type 2 diabetes, obesity, and metabolic syndromes. PTP1B attenuates growth hormone-mediated Jak2–Stat signaling, providing another possible mechanism for PTP1B roles in obesity [5]. 

Due to the key roles played by protein tyrosine phosphatases in cancer biology, they constitute promising targets for the development of new anti-cancer diagnostic and therapeutic strategies [6]. Researchers around the world are working on design studies of PTP1B inhibitors. Liu group proposed synthetized 4-thiazolinone derivatives, effective against PTP1B, with an the half maximal inhibitory concentration (IC_50_) of 0.92 μM [7]. Small molecular effective inhibitors targeting PTP1B have been identified by Jin’s group as series of compounds containing dihydropyridine thione [8].

Advancements have been made in PTP1B-related drug discovery utilizing compounds from natural products. Furthermore, an integrated strategy combining medicinal chemistry and structural biology will hopefully result in the design of potent and selective PTP1B inhibitors [9]. It has been discovered that flavonoids can act as effective protein tyrosine phosphatase inhibitors [10]. In our studies, we focused on natural compounds that can be administered with the diet. Our group has lately documented the effect of oleuropein, the phenylethanoid–phenolic compound found in the olive leaf, on PTP1B enzymatic activity [11]. Previous studies have shown that also fatty acids may be natural inhibitors of the similar pro-oncogenic tyrosine phosphatase SHP2 [12]. It is worth noting that fatty acids have recently played an important role in the design of treatments for numerous diseases, especially cancer. Our previous results confirmed that, in comparison to hydrogen peroxide, selected carboxylic acids, e.g. octanoic acid, possess a significantly higher binding affinity for PTPs active sites and can be strong inhibitors of PTPs, including PTP1B [13]. 

Omega-3 compounds are essential polyunsaturated fatty acids necessary for human health, which must be administered through diet. Omega-3 fatty acids, such as eicosapentaenoic (EPA) or docosahexaenoic (DHA) acids, are found in deep water fishes, for example, in mackerel, tuna, and salmon [14]. Cold-water oily fishes are the main dietary source of DHA for humans, providing relatively large amounts of DHA [15]. 

It was found that natural products, such as polyunsaturated fatty acids (PUFAs), are able to exert anticancer effects by affecting cell proliferation, metastasis, apoptosis, autophagy, and angiogenesis [16]. EPA and DHA acids were found to inhibit important angiogenic factors (platelet-derived growth factor, vascular endothelial growth factor (VEGF) and endothelial cell growth factor) and are effective against colorectal adenocarcinoma and breast cancer [17]. DHA and EPA, in the form of acid and phospholipids, have been shown to inhibit the viability of colorectal cancer cells. Furthermore, PUFAs have been shown to have stronger inhibitory effects on the growth of the HT-29 cell line than on the growth of Caco-2 and DLD-1 cells [18].

**Figure 1:**
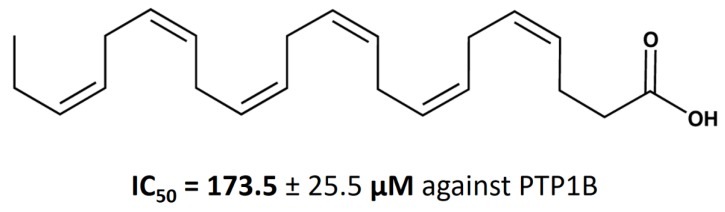
Structure of docosahexaenoic acid (DHA) with the calculated the half maximal inhibitory concentration (IC_50_) value for Protein tyrosine phosphatase 1B (PTP1B). The IC_50_ value was determined from a graph showing inhibitory concentration versus percentage of the enzymatic activity, presented as absorbance using para-nitrophenyl phosphate (*p*NPP) as the substrate of recombinant PTP1B during 30 min of incubation with DHA, at a substrate concentration equal to the Michaelis-Menton constant (Km value, the substrate concentration at which the half of the enzyme molecules are associated with substrate). Data were presented as an estimated concentration ± standard deviation in the graph.

Many studies have pointed to the potential value of omega-3 fatty acids as adjuvant therapy to standard chemotherapy, as it has been shown to enhance the potency of doxorubicin or mitomycin C in breast cancer cells [19,20]. The protective role of an omega-3 fatty acid-enriched diet was evidenced also against prostate and colon cancer [21,22]. Although the effect of DHA has already been studied in human cancer cell lines, such as MDA-MB-231, SiHa, Raji, DHL-4, and breast cancer MCF-7 [23], the impact of DHA against PTP1B involved in breast cancer development has never been evaluated. Here, we examined the inhibitory properties of DHA (Figure 1) against pro-oncogenic PTP1B. We also performed studies on the MCF-7 cell line to confirm the inhibitory effect of DHA on the viability of cells reported by other research groups [24,25]. 

## 2. Materials and Methods 

### 2.1. Recombinant PTP1B Activity Assay

A solution of recombinant PTP1B was prepared in 10 mM HEPES buffer pH 7.4, with a final concentration of PTP1B phosphatase in the reaction sample of 1.5 μg/mL (3.3 nM). The enzyme was untreated (control) or treated with DHA solutions in HEPES buffer. The concentrations of DHA presented in the figures below indicate the final concentration of DHA in the samples. The measurement was performed in 96-well microplates. The final volume of each reaction sample was 200 μL. The enzymatic activity of PTP1B was measured at 37 °C using the chromogenic substrate para-nitrophenyl phosphate (pNPP, 2 mM) by reading the solution absorbance at 405 nm with a microplate reader (Jupiter, Biogenet). DigiRead Communication Software (Asys Hitech GmbH, Eugendorf, Austria) was used to read the results. The reduction assay was performed using dithiotreitol (DTT). All reagents were from Sigma Aldrich.

### 2.2. Cell Culture 

MCF-7 cells were cultured in DMEM medium completed with 10% fetal bovine serum, 100 μg/mL penicillin/streptomycin, and 2 mM l-glutamine. The culture was maintained in an atmosphere containing 5% CO_2_ at 37 °C. The cell culture density was kept to a maximum of 1 × 10^6^ cells/mL. At least every two days, the medium was replaced with a fresh one, and the cells were counted and reseeded to new plates to maintain the recommended density.

### 2.3. Cell Viability Assay

The cells (1 × 10^6^ cells/mL) were untreated (control) or treated with solutions of DHA in 0, 1% dimethyl sulfoxide (DMSO). The concentrations of DHA presented in the figures below indicate the final concentration of DHA in samples. The control sample was treated with the same amount of 0, 1% DMSO solution. After the recommended incubation time, the cells were suspended in a solution of 5 mg/mL MTT (3-[4,5-dimethylthiazol-2-yl]-2,5-diphenyltetrazolium bromide) in DMEM in the absence of phenol red. Then, the samples (100 μL) were incubated for 3–4 h at 37 °C in 96-well plates. When a purple-colored precipitate was visible under the microscope, 100 μL of DMSO was added to each well, and the plate with a cover was left in the dark. After 15 min of incubation, the absorbance was determined at 590 nm using a microplate reader.

### 2.4. Computational Analysis

The DHA molecule was docked on selected binding sites of the enzyme to predict the binding conformation and supramolecular interactions. The initial structure of PTP1B was taken from the Research Collaboratory for Structural Bioinformatics protein data bank (www.pdb.org) with code 5K9V.pdb. Then, the structure was loaded into Molecular Operating Environment software (Chemical Computing Group, Montreal, Canada), and water molecules were removed. Polar hydrogen atoms were added. The structure was protonated at a temperature of 300 K, pH 7, and salt concentration of 0.1. Ligands were removed, and the structures were optimized using the Amber10: EHT force field software. The DHA molecule was also docked to the structure through an allosteric site. The side chains were kept free to move during force-field refinement. The placement method used with default settings was Alpha PMI. The top 30 docking modes were retained for DHA, and these poses were ranked by London dG scoring function to estimate the free energy of binding of peptide conformers. The lowest score pose (most stable pose) was chosen from the top conformation, and its binding orientation was used to calculate binding interactions.

### 2.5. Statistical Analysis

All the experiments were performed with at least three repetitions. The data were analyzed with GraphPad Prism Software v.4 (GraphPad Software, San Diego, CA, USA). Statistical analysis was performed utilizing the ANOVA test combined with Tukey’s test or the Student’s *t* test combined with Wilcoxon test. The data are presented as means ± SD. Differences between means were considered significant for *p* < 0.05.

## 3. Results

### 3.1. Inhibitory Effect of DHA on the Enzymatic Activity of PTP1B 

We examined the effect of DHA on the enzymatic activity of PTP1B. In order to estimate the inhibitory impact of DHA on PTP1B, we performed an enzymatic activity assay using recombinant PTP1B after treatment with the tested compound. 

**Figure 2:**
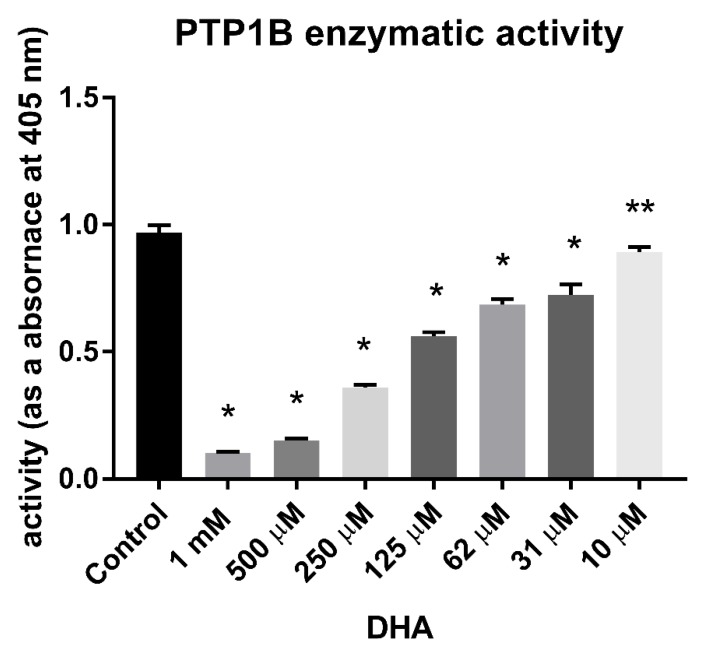
Enzymatic activity of PTP1B after 30 min of treatment with different concentrations of DHA. Data are presented as absorbance measured at 405 nm, means ± SD (*n* = 3). One-way Anova test. * Means were significantly different from the control values (*p* < 0.0001), ** (*p* < 0.001).

We found that DHA was able to decrease the enzymatic activity of PTP1B at concentrations in the micromolar range (Figure 2). The inhibition of the phosphatase was concentration-dependent. A concentration of 10 μM DHA induced only a slight effect on the enzymatic activity of PTP1B, while concentrations higher than 30 μM had a significantly inhibitory impact (Figure 2); concentrations of 500 µM and 1 mM induced a high level of inactivation of PTP1B (Figure 2).

**Figure 3:**
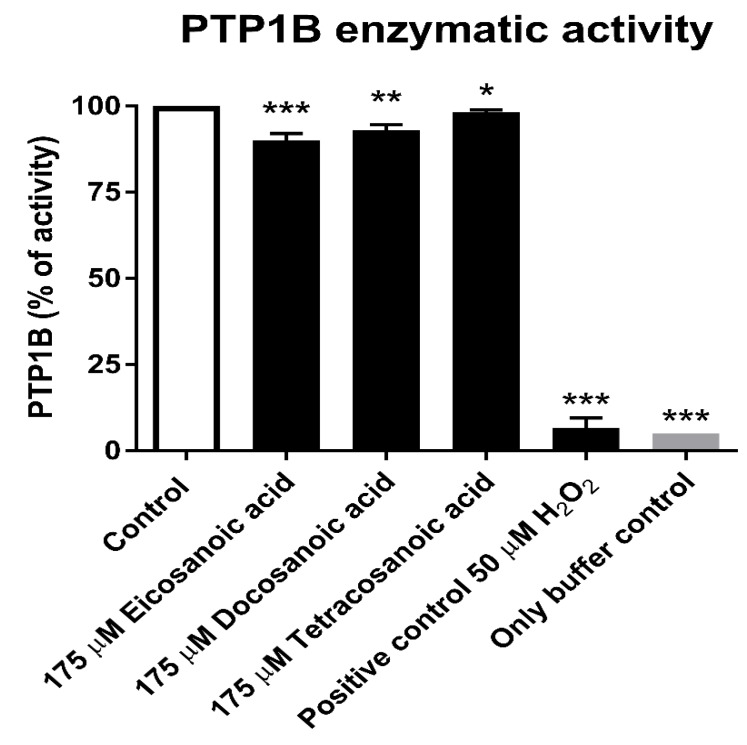
Enzymatic activity of PTP1B after 30 min of treatment with 175 µM eicosanoic, docosanoic, and tetracosanoic acids. The positive control was PTP1B treated with 50 µM H_2_O_2_, while the negative control was buffer only. Data are presented as absorbance measured at 405 nm, means ± SD (*n* = 3). One-way Anova test. * Means were not significantly different from the control values. ** Means were significantly different from the control values (*p* < 0.001), *** (*p* < 0.0001).

To compare the effect of DHA with those of other fatty acids, we performed additional PTP1B enzymatic activity analyses (Figure 3). We assessed the effect of similar fatty acids on PTP1B activity. We choose eicosanoic acid (which contains 20 carbons in a carbon chain), docosanoic acid (with 22 carbons), and tetracosanoic acid (with 24 carbons). All selected acids have carbon chains of length similar to that of DHA, but in contrast to DHA, they are saturated fatty acids. As we can observe in Figure 3, a concentration of 175 µM of the tested saturated acids had only a slight effect on PTP1B activity (decreasing it by only 2.2–10%); a concentration of 175 µM is the IC_50_ value of DHA for PTP1B (Figure 1). We showed that unsaturated DHA possessed a significantly higher inhibitory activity on PTP1B in comparison to similar saturated fatty acids. 

We performed the measurements including a positive control consisting of hydrogen peroxide, which is a known PTPs reversible inhibitor that induces complete PTP1B inactivation at a concentration of 50 µM (Figure 3).

### 3.2. Calculation of DHA IC_50_ Values for PTP1B 

To compare the impact of DHA on PTP1B with that of other phosphatase inhibitors, we calculated the respective IC_50_ values for PTP1B (Figure 1). We treated PTP1B with different concentrations of DHA for 30 min. Then, we calculated the IC_50_ values on the basis of a graph showing DHA concentration versus percentage of enzymatic activity of recombinant PTP1B, presented as absorbance using *p*NPP as the substrate. The *p*NPP substrate concentration for IC_50_ calculations was equal to the Km value determined for PTP1B; the Km value is defined as the substrate concentration at which enzyme activity is at half maximum. The obtained results indicated that DHA can decrease the enzymatic activity of PTP1B and is effective in a concentration range equal to 173.5 ± 25.5 µM (Figure 2).

### 3.3. Reduction Assay 

**Figure 4:**
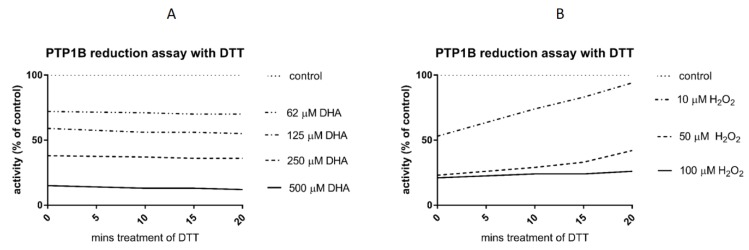
(**A**) Reduction assay of PTP1B, previously inhibited by DHA, in the presence of dithiotreitol (DTT). Data are presented as percent of the control activity (100% untreated PTP1B), means ± SD (*n* = 3). Student’s *t* test combined with Wilcoxon test. Means were not significantly different from each other (*p* > 0.05). (**B**) Reduction assay of PTP1B, previously inhibited by H_2_O_2_, in the presence of DTT. Data are presented as percent of the control activity (100% untreated PTP1B), means ± SD (*n* = 3).

A reduction assay was performed to estimate the reversibility of docosahexaenoic acid-induced PTP1B inactivation after addition of the thiol reducer DTT. The results showed that incubation with the thiol reducer for 20 min did not affect the inhibition of PTP1B by DHA. Figure 4 shows the effect of selected DHA concentrations. In our studies, the loss of activity of recombinant PTP1B caused by DHA could not be restored after incubation with 10 mM DTT (Figure 4A). We compared the results with those of the known reversible inhibitor of PTPs hydrogen peroxide and observed recovery of the hydrogen peroxide-inhibited enzyme activity after incubation with DTT (Figure 4B).

### 3.4. DHA Effect on the Viability of MCF-7 Breast Cancer Cells

**Figure 5:**
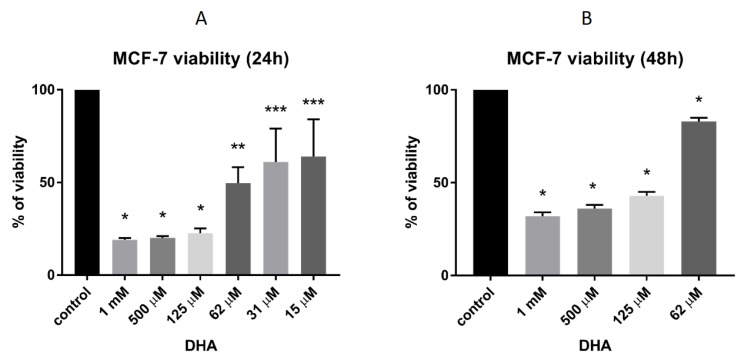
(**A**) Cell viability of MCF-7 breast cancer cells after treatment with different concentrations of DHA for 24 h. Data are presented as percent of the control (100% viability, untreated cells ), mean ± SD (*n* = 3). One-way Anova test combined with Tukey test. * Means were significantly different from the control values (*p* < 0.0001). ** Means were significantly different from the control values (*p* < 0.001). *** Means were significantly different from the control values (*p* < 0.01); (**B**) Cell viability of MCF-7 breast cancer cells after treatment with different concentrations of DHA for 48 h. Data are presented as percent of the control viability (100% viability, untreated cells), mean ± SD (*n* = 3). One-way Anova test combined with Tukey test. * Means were significantly different from the control values (*p* < 0.0001).

To evaluate the effect of DHA on the viability of breast cancer cells, we performed an MTT viability test using the MCF-7 cell line. We found that DHA could decrease the cell viability in a concentration-dependent manner. After 24 h of treatment, the viability of the cells was significantly (*p* < 0.0001) decreased by 150 μM DHA (Figure 5A). Even 15 μM DHA revealed an inhibitory effect (*p* < 0.01). To evaluate the impact of DHA on breast cancer cells after a longer exposition, we treated the cells for 48 h with DHA. We found that the DHA significantly (*p* < 0.0001) decreased the cell viability (Figure 5B), but we observed that after 48 h of incubation, DHA had a slightly lower inhibitory effect on MCF-7 cells than after a shorter exposure.

### 3.5. Docking of DHA to PTP1B 

**Figure 6:**
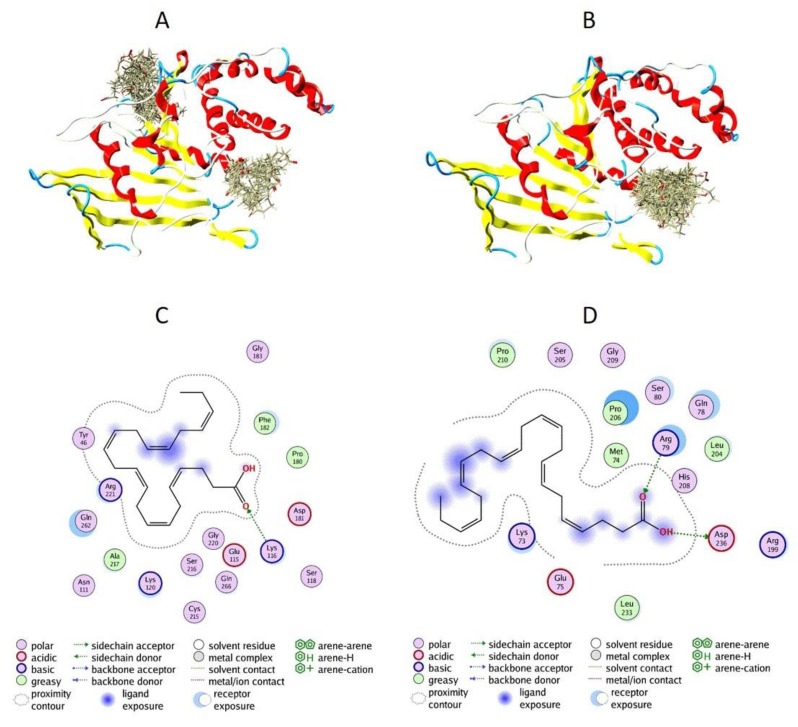
Computational analysis of DHA binding to PTP1B. (**A**) Blind docking of DHA with PTP1B. Top 30 binding conformations of DHA with the PTP1B molecule. (**B**) Allosteric site-directed docking of DHA with PTP1B. Top 30 binding conformations of DHA with the PTP1B allosteric site. (**C**) Binding interactions of DHA with the PTP1B allosteric site. (**D**) Binding interactions of DHA with the PTP1B binding site obtained from blind docking.

To evaluate the mechanism of PTP1B inactivation by DHA, we decided to utilize computational methods of inhibitor binding to PTP1B. We performed a docking analysis using blind docking in a first step (Figure 6A), followed by allosteric site-directed docking (Figure 6B). The blind docking results allowed us to assume that DHA is able to bind to two binding sites, including the allosteric site (Figure 6A). The allosteric site-directed docking showed that all the top 30 conformations of DHA could bind to the allosteric site (Figure 6B). To evaluate the binding conformations and possible interactions of DHA with each of the binding sites, we performed additional computational analysis. We found that when DHA is bound to the allosteric site, it is probably interacting with Lys116, Tyr46, and Arg221 (Figure 6C). We identified possible interactions between DHA and Arg79 as well as Asp236 in the second binding site (Figure 6D).

## 4. Discussion

Breast cancer is known to be one of the most common female cancers. It most frequently presents multiple organ metastases and has a complicated etiology. In spite of therapeutic advances, the number of breast cancer cases has still been growing in recent years [26].

Recent studies demonstrated that DHA and its analogs can have a significant effect on cancer metabolism and can be considered for use in chemotherapy. Numerous studies showed that marine omega-3 consumption is associated with a lower risk of breast cancer [27,28,29,30]. The ability of omega-3 fatty acids, such as DHA and EPA, to induce cytotoxicity via apoptosis in many cancer cell lines was previously discovered. The omega-3 fatty acids were shown to potentially target multiple molecular signaling pathways involved in cancer cell death [31]. Importantly, DHA was shown to suppress cell proliferation and increase apoptosis in breast cancer cell lines [32,33,34]. Moreover, increased intake of omega-3 acids, including DHA, through the diet is considered to be effective not only for the prevention but also for the treatment of breast cancer [33]. Furthermore, DHA supplementation during chemotherapy improved docetaxel efficacy in patient-derived breast cancer xenograft models [35]. Importantly, dietary DHA may also limit the adverse effects of standard chemotherapeutics. It was found to play a protective role against neuroinflammation and synaptic damage induced by chemotherapy utilizing doxorubicin [36]. Therefore, DHA may constitute an important tool in novel adjuvant breast cancer therapy. In our studies, we confirmed that DHA is able to decrease the viability of estrogen receptor-positive MCF-7 breast cancer cells.

It has been previously shown that selected fatty acids reduced the activity of the pro-oncogenic phosphatase SHP2 [12]. In the present paper, we investigated the effect of selected omega-3 fatty acids on PTP1B which, together with SHP2, is implicated in breast cancer development. Notably, it was found that PTP1B is directly implicated in carcinogenesis of estrogen receptor-positive breast cancer [37]. Moreover, PTP1B was found to upregulate the proliferation and inhibit the death of both HER2-positive and triple-negative breast cancer cells [38].

Protein tyrosine phosphatases are sensitive to oxidation, as oxidation caused by, e.g., hydrogen peroxide leads to reversible conversion of the catalytic cysteine to a sulphenic acid residue [39]. The enzymatic activity can be restored by addition of a thiol reducer such as DTT. In our studies, we showed that DHA is able to inhibit PTP1B and that the reduction of the thiol group did not reverse the inactivation. This proves that the inactivation of PTP1B by DHA does not involve the oxidation of the cysteine residue in the phosphatase active site, which is the main mechanism of inactivation of protein tyrosine phosphatases [40]. We compared the effect of DHA with those of similar fatty acids, i.e., eicosanoic acid (with 20 carbons in the carbon chain), docosanoic acid (22 carbons), and tetracosanoic acid (24 carbons). All those acids, in contrast to DHA, are saturated fatty acids. We found that those acids had only a slight effect on PTP1B activity in comparison to DHA. This may be an interesting observation, as DHA was discovered to be able to form helical conformation unusual for fatty acids [41]. It cannot be excluded that this property allows the DHA molecule to specifically interact with the binding site of PTP1B.

Our computational analysis showed that DHA is able to bind to different binding sites of PTP1B, including the allosteric site. We found that when DHA is bound to the allosteric site, it is probably interacting with Arg221 (Figure 6C). The arginine residue (Arg221 in PTP1B) plays is important for substrate binding and stabilization of the transition state [42]. Together with Asp181, it mediates the closure of the conserved protein loop (WPD loop), essential for catalysis. The hydrophobic WPD loop environment consists of three conserved residues (Tyr176, Trp179, and Arg221) [43]. Mutations found in Yersinia PTP revealed that the corresponding residues of Tyr176, Trp179, and Arg221 may be implicated in the allosteric control of PTP enzymatic activity [44]. The enzymatic mechanism of protein dephosphorylation is involved in the binding of a phosphotyrosine substrate to PTP1B and in the promotion of a conformational change in the WPD loop. The corresponding loop moves closer to the phosphotyrosine and allows the side chain of the Asp181 residue to act as a general acid/base. The side chain of Arg221 changes orientation and coordinates the closure of the WPD loop. Interactions between Arg221 and Trp179, which stabilize the phosphate group, therefore stabilize the WDP loop in the closed conformation [45]. 

## 5. Conclusions

In conclusion, using both experimental and computational methods, we were able to confirm that DHA can decrease the viability of MCF-7 breast cancer cells, as well as the enzymatic activity of pro-oncogenic PTP1B. We showed that unsaturated DHA possessed a significantly higher inhibitory activity toward PTP1B in comparison to similar saturated fatty acids. Our studies also revealed that DHA can bind to the PTP1B allosteric site and interact with the residues directly responsible for phosphatase activity. Utilizing both a recombinant enzyme and cellular models, we demonstrated that DHA can be regarded as a potential pharmacological agent in the treatment or prevention of breast cancer. For example, it can be initially utilized as a support to adjuvant therapy. The analysis of the interactions of DHA with the allosteric site of PTP1B could also be useful in future inhibitor/drug design studies.
